# Analysis of Complex Absorption After Multiple Dosing: Application to the Interaction Between the P-glycoprotein Substrate Talinolol and Rifampicin

**DOI:** 10.1007/s11095-022-03397-6

**Published:** 2022-09-26

**Authors:** Michael Weiss, David Z. D’Argenio, Werner Siegmund

**Affiliations:** 1 Department of Pharmacology, Martin Luther University Halle-Wittenberg, Halle, Germany; 2 Department of Biomedical Engineering, University of Southern California, Los Angeles, CA USA; 3 Department of Clinical Pharmacology, Center of Drug Absorption and Transport (C_DAT), University Medicine Greifswald, Greifswald, Germany

**Keywords:** absorption kinetics, interaction, multiple dosing, rifampicin, talinolol

## Abstract

**Purpose:**

In order to clarify the effect of rifampicin on the bioavailability of the P-glycoprotein substrate talinolol, its absorption kinetics was modeled after multiple-dose oral administration of talinolol in healthy subjects.

**Methods:**

A sum of two inverse Gaussian functions was used to calculate the time course of the input rate into the systemic circulation.

**Results:**

The estimated rate of drug entry into the systemic circulation revealed two distinct peaks at 1 and 3.5 h after administration. Rifampicin did not affect bioavailability of talinolol, but did shift the second peak of the input function by 1.3 h to later times. Elimination clearance and one of the intercompartmental distribution clearances increased significantly under rifampicin treatment.

**Conclusions:**

Rifampicin changes the time course of absorption rate but not the fraction absorbed of talinolol. The model suggests the existence of two intestinal absorption windows for talinolol.

**Supplementary Information:**

The online version contains supplementary material available at 10.1007/s11095-022-03397-6.

## Introduction

Although the oral route is the most convenient and most used method of drug delivery, pharmacokinetic modeling is often based on noncompartmental methods (numerical integration) or oversimplified models like the first order absorption model. For the more complex absorption behaviors of extended release formulations or to study food effects, flexible empirical input rate models, such as the inverse Gaussian density function [1–5], or a sum of inverse Gaussian functions [6–10], have been successfully applied. The latter was capable of fitting double-peak data [11] and has some advantages over models based on transit compartments (gamma density) or a Weibull function [8]. All of the aforementioned studies involve applications of absorption models to single dose data.

In this work, we describe the use of the sum of two inverse Gaussian functions (2IG) as oral input rate function in a multiple dosing situation, namely after repeated oral administration of talinolol. Talinolol is a substrate of the efflux transporter P-glycoprotein (P-gp) that has been extensively used to investigate P-gp-mediated drug transport [12, 13] and is recommended by FDA as a probe substrate. The pharmacokinetics of talinolol was evaluated before and after rifampicin administration in order to analyze the effect of P-gp induction on the disposition and absorption of talinolol. In contrast to the previous analysis that was based on noncompartmental methods [14], the approach we present provides an estimate of the rate of talinolol absorption.

Thus, the first purpose was to estimate the time course of rate of talinolol absorption, to determine the effects of rifampicin which remained undetected when using noncompartmental analysis. Our reevaluation of the data by Westphal *et al*. [14] suggests that rifampicin-mediated P-gp induction affects the intestinal site of talinolol absorption but not bioavailability. Furthermore, reasons for the observed double-peak phenomenon [15] can now be interpreted in terms of the absorption rate profile. Our results shed new light on the role of P-gp–mediated intestinal transport of talinolol and the role of the P-gp-inducer rifampicin [16–18]. A second purpose of this study was demonstrate how multiple dose data can be analyzed with the ADAPT 5 software [19] using a complex absorption model.

## Methods

### Clinical Study Data

We reevaluated the pharmacokinetic data of a previously performed repeated dose drug-drug interaction study with talinolol and rifampicin [14]. Healthy human subjects were treated with talinolol for 14 days (100 mg/day, 07:00 h a.m.). Beginning with the 9^th^ treatment day, rifampicin (600 mg/day, 06:00 h p.m.) was co-administered for 9 days. Concentrations-time curves of talinolol during repeated-dosing were measured at the 7^th^ and 14^th^ treatment day. 8 days before and 3 days after the last oral treatment with talinolol, the serum concentration–time curves of the drug were measured after short-time intravenous infusion (30 mg within 30 min). The data were stored in our databank and used for re-evaluation by pharmacokinetic modeling in fully anonymized manner in agreement with the written informed consent as given by the healthy subjects included in the study (8 males, age 22—26 years; body weight 67–84 kg). The study had been approved by the Independent Ethics Committee of the University Medicine of Greifswald.

### Pharmacokinetic Modeling

The time course of the absorption rate (rate of drug input into the central compartment), *I*(*t*) was described as a sum of two IGs [7, 10, 11]

1 $$\begin{array}{cc}I\left(t\right)=DF\left({pf}_{1}\left(t\right)+\left(1-p\right){f}_{2}\left(t\right)\right)& 0<p<1\end{array}$$

where *D* is dose, *F* is bioavailability, *f*_*i*_(*t*) denotes the IG function below and *p* is a nonnegative quantity that defines the relative contribution of each IG to the input function *I*(*t*).

2 $${f}_{i}(t)=\sqrt{\frac{M{T}_{i}}{2\pi \text{\hspace{0.05em}}R{D}_{i}^{2}\text{\hspace{0.05em}\hspace{0.05em}}{t}^{3}}}\mathrm{exp}\left[-\frac{{(t-M{T}_{i})}^{2}}{2\text{\hspace{0.05em}}R{D}_{i}^{2}\text{\hspace{0.05em}\hspace{0.05em}}M{T}_{i}t}\right],\text{\hspace{0.17em}\hspace{0.17em}\hspace{0.17em}\hspace{0.17em}}t>0$$

where *MT*_*i*_ and $R{D}_{i}^{2}$ are the scale and shape parameters, respectively, of the *i*th IG function. The mean input time (MIT) is then given by

3 $$MIT=pM{T}_{1}+(1-p)M{T}_{2}$$

This two IG input model above can be extended to represent a mixture of multiple IG. For multiple doses, the input function can be written as:

4 $$I(t)=\sum_{j=1}^{ND}\left({D}_{j}F(p{f}_{1}(t-d{t}_{j})+(1-p){f}_{2}(t-d{t}_{j})\right)$$

where ${D}_{j}$ and $d{t}_{j}$ are the amount and time of the jth dose, and ND is the number of doses.

The disposition of talinolol, both with and without rifampicin, was described using linear compartment models.

### Parameter Estimation

A stepwise estimation process was followed. First, the intravenous (iv) data obtained from the experiments with and without rifampicin were each analyzed using two and three compartment models. From these results, the disposition parameters of the resulting three compartment model were fixed, and the parameters of the absorption model (input to the central compartment) were then estimated from the respective concentration time data of talinolol after oral administration. Note that six parameters, namely *F, MT*_*1*_*,*$R{D}_{1}^{2}$*, MT*_*2*_*,*$R{D}_{2}^{2}$ and *p* were estimated from the oral data. Both estimation steps were performed by population analysis (nonlinear mixed-effects modeling) using the maximum likelihood expectation maximization (MLEM) application in the ADAPT (Version 5) software [19]. See Supplemental Material for the code of the ADAPT file implementing the model. All model parameters (compartment disposition model and inverse Gaussian mixture model) were assumed to follow a multivariate log-normal distribution. The residual errors for both the iv and oral modeling analyses were assumed to be normally distributed with proportional and additive variance terms. Model selection (number of compartments in the disposition model) was based on the likelihood-derived Akaike information criterion (AIC). Plots of conditional standardized residuals and the relative standard errors of estimated parameters were also examined.

For the visual predictive check, plasma concentration *versus* time profiles were simulated for 1000 virtual subjects, using the estimated parameter mean values and variances (Monte Carlo simulation, population simulation without process noise). Lognormal parameter distributions were assumed. The 5th percentile, 50th and 95th percentile of model-predicted concentrations at each time point were extracted from the simulated data.

From the estimated parameters describing the time course of talinolol absorption before and during coadministration of rifampicin, the time points of the first and second peak and the maximum input rates were calculated using the statistical program package Maple (https://de.maplesoft.com). This software was also used to simulate the time course of the amount absorbed with the cumulative IG [20]. The differences between pre- and post-rifampicin treatment were analyzed by the paired t-test. We evaluated correlations between I_max,1_ and t_max,1_ and between t_max,2_ and t_max,1_ using linear regression analysis.

## Results

**Fig. 1:**
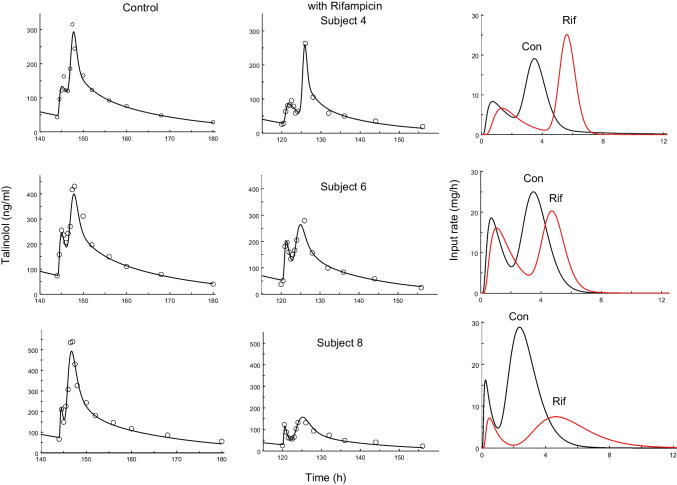
Fits of talinolol data after 100 mg/24 h talinolol in 3 subjects before (left) and after coadministration of rifampicin (middle). The resulting time courses of input rate are shown on the right hand side. The first dose was given at Time 0.

**Fig. 2:**
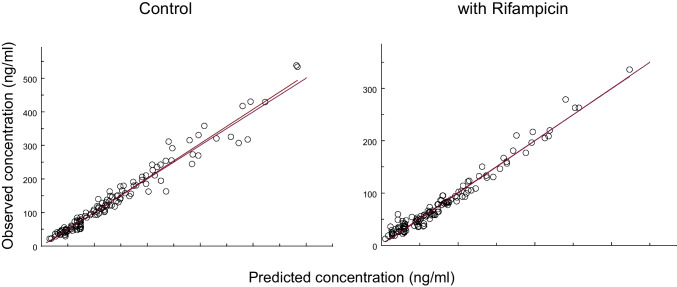
Goodness-of-fit plots showing the observed concentration data of talinolol after multiple oral dosing *versus* the individual model-predicted values for all 7 subjects without (left) and with rifampicin (right). The regression lines are nearly coincident with the lines of identity.

**Fig. 3:**
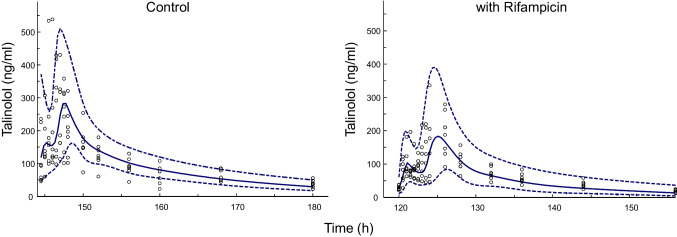
Visual predictive checks showing the 90% prediction interval (5th to 95th percentiles, dashed lines) and the median on the simulated data (solid line) together with the observed data (open dots).

**Fig. 4:**
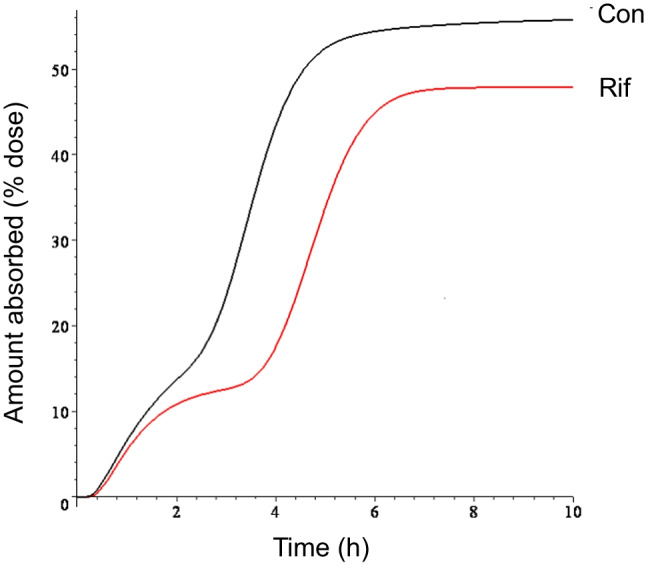
Mean cumulative amount of talinolol absorbed as fraction of dose. The curves were simulated using the mean parameter estimates of the input function.

**Fig. 5:**
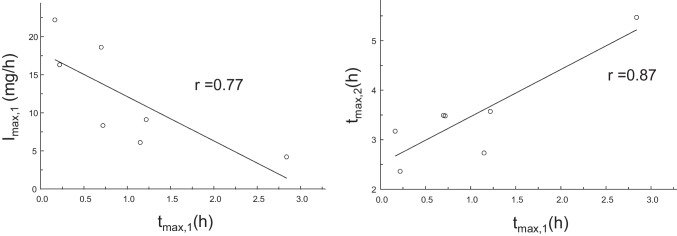
The first peak of the input rate decrease linearly (*p* < 0.05) with the time at which peak concentrations are reached (left) and the time point of the second peak increases with that of the first peak (*p* < 0.05) (right).

Talinolol iv data were best described (AIC criterion) by a 3-compartment model (Table 1). Rifampicin caused a significant increase in both elimination clearance and intercompartmental clearance 1 (*p* < 0.01). Renal clearance remained unchanged. Inspection of the oral data indicated a mixture of two IG functions would be appropriate for each subject. Our multiple dose approach with a 2IG input model allowed an excellent fit to all talinolol serum concentration data, obtained before and after coadministration of rifampicin. In one subject, the model could not be fitted to the data because of an unexplained discrepancy between the trough and peak concentration. This subject was excluded during the analysis. The quality of fit is illustrated for three subjects with prototypical time courses of input rate in Fig. 1, by the goodness of fit plots (Fig. 2) and a visual predictive check (Fig. 3). The input function (Fig. 1) reflects the characteristic double peak phenomena observed in the serum concentration–time curves. No significant changes in the parameters of the input function were detected in the presence of rifampicin, except for an increase in the time at which the second peak appears, namely from 3.47 to 4.74 h (*p* < 0.05). A shift to the right of the second peak was observed in all subjects, albeit with varying degree (Fig. 1). The mean bioavailability of talinolol was not significantly changed by rifampicin, despite a considerable reduction in two subjects (e.g. subject 8 in Fig. 1). The difference in the time courses of the cumulative amount absorbed are clearly visible in Fig. 4. Interestingly, the maximum of the first peak, I_max,1_, decreased with increasing t_max,1_ (*p* < 0.05) and there was a significant correlation between t_max,2_ and t_max,1_ (*p* < 0.05) (Fig. 5). All these correlations disappeared under rifampicin.

**Table 1:** Pharmacokinetic parameters after a single intravenous dose (30 mg) and multiple oral dosing (100mg/24h) of talinolol without and with rifampicin in 7 healthy human subjects (population means with intersubject variability, %CV)

		Control		With Rifampicin	
Model parameter	Symbol (Unit)	Mean	Inter CV	Mean	Inter CV
Intravenous dose			(%)		(%)
Clearance	CL (mL/min)	320	10	420**	20
Volume of central compartment	V_c_(L)	15.6	18	16.8	19
Intercompartmental clearance 1	CL_p1_ (mL/min)	892	22	1337**	29
Volume of peripheral compartment 1	V_p1_ (L)	87.8	29	104	41
Intercompartmental clearance 2	CL_p2_ (mL/min)	457	17	408	45
Volume of peripheral compartment 2	V_p2_ (L)	188	13	40.1	36
Steady-state distribution volume	V_ss_ (L)	291	13	293	17
Residual variability^a^	s_0_	0.19		5.5	
	s_1_	0.1		0.3	
Oral dose					
Bioavailability	F (%)	56.2	18	48.4	28
Mean input time	MIT (h)	3.13	28	3.93	20
Time of the first peak	t_max,1_ (h)	1.00	90	0.82	52
Input function at t_max,1_	I_max,1_ (mg/h)	12.1	57	10.2	32
Time of the second peak	t_max,2_ (h)	3.47	29	4.74*	21
Input function at t_max2_	I_max,2_ (mg/h)	22.2	26	20.2	46
Residual variability^a^	s_0_	2.1		4.6	
	s_1_	0.13		0.07	

^a^Residual error has a variance: *VAR*_*i*_ = [s_0_ + s_1_*C*(*t*_i_)]^2^

*p < 0.05

**p < 0.01

## Discussion

The modeling approach used in this work provides an estimate of the time course of talinolol’s rate of absorption under multiple dosing, an advantage compared to previously used noncompartmental analysis, which allowed us to quantify the effects of rifampicin on talinolol’s absorption. From our modeling analysis, the double peak in plasma concentrations is obviously due to a bimodal pattern of input rate. Note that we did not observe a decrease in bioavailability in contrast to the initial noncompartmental evaluation by Westphal *et al*. 2000 [14], who erroneously used iv control instead iv treatment data as previously noted by Chiou *et al*. 2003 [21]. This correction, however, has been overlooked in later publications citing the original article [16, 22]. Thus, coadministration of rifampicin neither influenced the amount of talinolol absorbed significantly, nor the mean absorption time; solely the second absorption peak was shifted to later times, ie, from 3.47 to 4.74 h (Figs. 1 and 4). The heights of both peaks did not change under rifampicin. The dip of the curve under rifampicin nearly coincidences with the second peak under control conditions. In other words, just at the time point when the input rate is maximal under control conditions, it is minimal after rifampicin.

What remains to be explained, however, are the processes underlying the first peak (after about one hour), which is not affected by P-gp induction, and the second higher peak (between 2 and 4 h after administration), which is shifted to later times after P-gp induction. Here we offer an alternative hypothesis to the “intestinal storage model” originally suggested by Weitschies *et al*. 2005 [15], which is based on more recent results on the abundance of multidrug transporters along the small and large intestine [23–25]. Intestinal P-gp is a high-affinity efflux carrier which counteracts the uptake. Therefore, the highest input-rates are expected in regions with lowest P-gp abundance and carriers saturated with the substrate talinolol. This occurs in the duodenum/jejunum and cecum/ascending colon where the P-gp abundance is lower compared to more distal regions [24] and indicates the existence of two absorption windows for talinolol. The first peak of the input rate occurred in about one hour after oral dosing, i.e., immediately after gastric emptying of water swallowed for drug administration in fasting healthy subjects [26]. Thus, t_max,1_ is not much different from the meta-mean of the gastric transit time in the fasted state of 1.37 h [27]. The large intersubject variability is due to one subject with a high t_max,1_ value of 2.84 h (and a low I_max,1_ of 4.20 mg/h, cf. Figure 5). The high variability of gastric emptying has been discussed elsewhere [28].The emptied water undergoes rapid absorption which generates high drug concentrations in the proximal small intestine [28, 29]. Therefore, the first peak is most likely caused by uptake in the duodenum/proximal jejunum and is limited by the relative short residence time in this region. Thus, the reduction of the first peak with increasing values of t_max,1_ (Fig. 5) may reflect the decrease in drug uptake after passing through the absorption window. Although only ~ 10% of the dose is absorbed from the proximal absorption window (Fig. 4), there is a significant correlation between F and I_max,1_ (r = 0.68, *p* < 0.01). Another ~ 40% is subsequently absorbed from a second absorption window with a maximum input rate after more than 3 h (Fig. 1). The correlation between F and I_max,2_ was less expressed (r = 0.60) but also significant (*P* < 0.05). This second window can likely be located to the cecum/ascending colon for the following reasons: Firstly, the P-gp abundance in the cecum/ascending colon is many times lower than in the ileum. Secondly, the time of maximum input rate was similar to the oro-cecal transit time as assessed with the sulfasalazine/sulfapyridine method (3 – 6 h) under identical protocol conditions [30, 31]. Interestingly, our estimate of 3.47 h is nearly identical with the meta-mean small intestinal transit time of 3.49 h [27]. Variability in oro-cecal transit time may be mainly due to the variability in gastric transit time, since the time point of the second peak increases linearly with that of the first peak (Fig. 5).

Regarding the effect of rifampicin on the absorption process, it appears that the first peak remained unaffected due to the relatively high, P-gp saturable talinolol concentration. P-gp inducing concentrations are also expected in the cecum/ascending colon by entero-hepatic recirculation of rifampicin after glucuronide cleavage [32]. However, talinolol concentrations are much lower than in the proximal small intestine. Thus, P-gp induction may avoid uptake in cecum/ascending colon leading to a shift of the second absorption window to a more distal site, with a delay of the second peak time by 1.27 h. This is based on the assumption that in deeper parts of the colon P-gp cannot be induced because the systemic concentrations after 600 mg rifampicin daily are too low to induce P-gp function [14, 33].

It remains unclear why the expected reduction in bioavailability is seen just in subjects 5 (F = 0.30) and 8 (F = 0.34, Fig. 1), i.e., in two out of seven subjects. These low values are the reason for the observed tendency to a bioavailabiity reduction (Table 1, Fig. 4).

The increase in talinolol clearance after rifampicin can be attributed to an increase of Pgp mediated intestinal secretion [14, 17], but we have no explanation for the significant increase in distribution clearance (Table 1). While a rifampicin mediated inhibition of P-gp in capillary endothelium of certain organs (e.g. brain, testes) [34] could increase tissue distribution, the intercompartmental clearance 1 exceeds blood flow to relevant organs. Thus we leave it as an open question for future research.

It should be added that physiologically-based pharmacokinetic modeling was used to predict rifampicin-mediated drug interaction [35] including talinolol [36, 37]. These modeling results suggested that this interaction can be explained by an increase of intestinal P-gp activity, which is in accordance with our hypothesis.

## Conclusions

These results show that more detailed information about the absorption process cannot be obtained from global parameters like bioavailability and mean absorption time. If they remain unchanged under coadministration of a P-gp inductor, this does not necessarily mean that drug absorption is not affected. By evaluating the time course of the input rate, we can get more insight into the region-specific absorption of talinolol. The results clearly demonstrate the advantages of our approach compared to the noncompartmental analysis.

## Supplementary Information

Below is the link to the electronic supplementary material.

Supplementary file1 (TXT 9.43 KB)

## Abbreviations

$R{D}_{i}^{2}$: Shape parameter of the *i*th inverse Gaussian function
*D*: Dose
*F*: Bioavailability
*f*_*i*_(*t*): *i*Th inverse Gaussian function
*I*(*t*): Input (absorption) rate
IG: Inverse Gaussian function
*I*_*max*__*,1*_: Maximum of the first peak of the input rate
*I*_*max*__*,2*_: Maximum of the second peak of the input rate
*MIT*: Mean input time
*MT*_*i*_: Scale parameter of the *i*th inverse Gaussian function
*ND*: Number of doses
P-gp: P-glycoprotein
*t*_*max,*__*1*_: Time of the first peak
*t*_*max,*__*2*_: Time of the second peak
